# Prognostic significance of lymphocytic foci composition in minor salivary gland biopsies for severe disease flare and severity in Sjögren’s syndrome: a 3-year follow-up cohort study

**DOI:** 10.3389/fimmu.2024.1332924

**Published:** 2024-02-26

**Authors:** Hye-Sang Park, Laura Martínez-Martínez, Berta Magallares López, Ivan Castellví, Patricia Moya, Helena Codes-Mendez, Nerea Hernandez Sosa, Cesar Diaz-Torne, Ana Laiz, Luis Sainz, Jose Luis Tandaipan, Anaís Mariscal, Teresa Franco-Leyva, Jordi Casademont, Candido Juarez, Hector Corominas

**Affiliations:** ^1^ Functional Unit of Systemic Autoimmune Diseases (UFMAS), Rheumatology Department, Hospital de la Santa Creu i Sant Pau, Biomedical Research Institute (IIB Sant Pau), Universitat Autònoma de Barcelona, Barcelona, Spain; ^2^ Functional Unit of Systemic Autoimmune Diseases (UFMAS), Immunology Department, Hospital de la Santa Creu i Sant Pau, Biomedical Research Institute (IIB Sant Pau), Universitat Autònoma de Barcelona, Barcelona, Spain; ^3^ Functional Unit of Systemic Autoimmune Diseases (UFMAS), Internal Medicine Department, Hospital de la Santa Creu i Sant Pau, Biomedical Research Institute (IIB Sant Pau), Universitat Autònoma de Barcelona, Barcelona, Spain

**Keywords:** Sjögren’s syndrome, immunofluorescence staining, fluorescent antibody technique, lip biopsy, minor salivary gland biopsy, histopathology, lymphocyte infiltration, lymphoid organization

## Abstract

**Introduction:**

This was an ambispective cohort study evaluating the prognostic significance of lymphocytic foci and its lymphoid composition in minor salivary gland biopsy (MSGB) for short-term disease flare and severity in Sjögren’s syndrome (SS).

**Methods:**

The inclusion criteria comprised individuals meeting the ACR/EULAR 2016 criteria who underwent MSGB with an infiltration of more than 50 lymphocytes and received clinical diagnosis between September 2017 and December 2018. Patients with inadequate biopsy samples were excluded. The number of lymphocytic foci and their lymphoid composition in MSGB were assessed using immunofluorescence staining. Major organ damage and improvements in the EULAR Sjögren’s Syndrome Disease Activity Index (ESSDAI) were measured. Statistical analyses, including Cox and linear regressions, were conducted.

**Results:**

A total of 78 patients with at least one lymphocytic focus were included in the study. The presence of higher T-cell counts in lymphocytic foci in MSGB was associated with severe disease flare, and a logarithmic transformation of T-cell count indicated increased risk (HR 1.96, 95% CI 0.91-4.21). Improvements in the ESSDAI were associated with higher total lymphocyte count and T- and B-cell numbers in the lymphoid composition of the lymphocytic foci. Seropositive patients exhibited higher T CD4+ cell numbers. Correlation analysis showed negative associations between age and lymphocytic foci and the T-cell count. Positive correlations were observed between antinuclear antibody (ANA) titers and total lymphocyte numbers.

**Discussion:**

Patients with a higher number of T cells in the lymphocytic infiltrates of lymphocytic foci may have a two-fold risk of severe disease flare. The number of B cells and T CD4+ cells in the lymphocytic infiltrates of lymphocytic foci showed a weak but positive relation with the ESSDAI improvement during follow-up. Age and seropositivity appeared to influence the lymphoid composition of the lymphocytic foci.

## 1 Introduction

Sjögren’s syndrome presents a diagnostic challenge due to the lack of a definitive gold standard test and the diverse range of clinical manifestations. The 2016 classification criteria for Sjögren’s syndrome aims to facilitate earlier detection in young patients with systemic symptoms. This is crucial for identifying candidates for clinical trials and introducing new therapeutic options. The immunological profile now focuses solely on anti-Ro antibodies, excluding antinuclear antibodies, rheumatoid factor, and isolated anti-La due to concerns about specificity, deviating from previous criteria (1). The diagnosis relies on the minor salivary gland biopsy (MSGB) since 20-40% of the patients are Ro/La antibody negative (2–5). It has proved to be especially useful when seronegative patients present with high clinical suspicion (6, 7). The results of the MSGB in these patients show a distinct feature consisting of a focal lymphocytic sialadenitis (FLS) characterized by the presence of one or more inflammatory infiltrates of at least 50 cells present in 4mm2 of gland surface (8, 9). Macrophages, dendritic cells, and natural killer cells are also present in a smaller proportion.

Traditionally, the focus score (FS), based on the quantification of lymphocytic foci in the MSGB (10), has been widely employed for diagnostic purposes, but it is frequently failed to be applied correctly in clinical settings due to miscalculation and difficulties in interpretation if other histopathologic alterations are present, and it fails to include important considerations such as the size of infiltrates in the foci (11, 12). It was also found that interobserver reliability was good, but there were disparities between assessment methods used by local pathologists (13). According to a retrospective study, the percentage of MSGBs with a focus score higher than one increased from 63% to 83% when using immunohistochemical staining, allowing the highlighting of small clusters of lymphocytes hardly visible when laying among the salivary ducts or acini (14).

At our institution, MSGB evaluations occur in the immunology department, involving a meticulous process overseen by two independent evaluators and an expert immunologist. Sequential biopsy sections are completely devastated for lymphocytic foci using immunofluorescence staining, with a comprehensive description of lymphoid composition. To ensure precision, only lymphocyte aggregates containing over 50 cells are considered foci, validating Sjögren’s syndrome diagnoses and minimizing false positives. This criterion was met by all patients, each presenting at least one lymphocytic focus. Immunofluorescence staining, by enabling the visualization and quantification of specific cellular components and lymphoid composition within salivary gland tissue, may improve diagnostic accuracy and discrepancy between observers. It permits the detection of well-circumscribed mononuclear inflammatory cell infiltrates (>50 cells), exhibiting a tightly packed dark zone and a more loosely packed light zone within salivary gland epithelium. Lymphoid composition and lymphocytic foci are believed to hold prognostic value for disease evolution and severity, yet evidence remains inconclusive.

The objective of this study is to assess the risk between significant disease flare and the lymphoid composition of the lymphocytic foci in the MSGB among Sjögren’s Syndrome patients over a 3-year follow-up period. The findings of this study will shed light on the potential value of immunofluorescence staining as a diagnostic technique with practical implications for clinical practice.

## 2 Materials and methods

### 2.1 Participants

This ambispective cohort study took place at the Systemic Autoimmune Disease Outpatient Clinic in the Rheumatology Department of the Hospital Universitari de la Santa Creu i Sant Pau, Barcelona, a regional referral unit for rare diseases and a healthcare provider for 450,000 AIS Dreta district residents under universal health coverage.

Patients aged 18 or older were screened for Sjögren’s syndrome diagnosis confirmation, primarily due to glandular or extraglandular organ symptoms with or without serological biomarkers. They underwent clinical assessments, including serological tests, by expert rheumatologists in the autoimmune disease clinic. Those with high clinical suspicion proceeded to routine clinical practice minor salivary gland biopsies (MSGBs) at the plastic surgery department.

Inclusion criteria required MSGBs with over 50 lymphocytes and compliance with the 2016 EULAR/ACR classification criteria, alongside clinical diagnosis by treating rheumatologists. Patients with inadequate biopsy samples were excluded. Recruitment spanned from September 1, 2017, to December 31, 2018, with data collection concluding in May 2022 after a 3-year follow-up period.

Patients were regularly monitored every 3-12 months based on disease severity, with data recorded until outcome onset, death, loss, or the observation period’s end.

### 2.2 Variables

We collected demographic data and data on the presence of other systemic autoimmune diseases (rheumatoid arthritis, systemic lupus erythematosus, systemic sclerosis, idiopathic inflammatory myopathies, antiphospholipid syndrome, or undifferentiated connective tissue disease). At each visit, we recorded the ESSDAI, concurrent use of corticosteroids, conventional or biologic disease-modifying anti-rheumatic drugs (DMARDs), and serological variables, including complement levels, immunoglobulins (IgA, IgM, and IgG), antinuclear antibodies, anti-Ro/SSA, anti-La/SSB, and rheumatoid factor (RF). Electronic medical records from the hospital were used for data collection.

Variables related to the lymphoid composition of the MSGB were the number of lymphocytic foci, the number of total lymphocytes, T cells, B cells, CD4+ cells, CD8+ cells, and the presence of inflammatory infiltrate that does not form lymphocytic foci.

### 2.3 Outcome

The primary outcome of this study was to evaluate if the number of lymphocytic foci and the lymphoid composition of the MSGB are prognostic factors for severe disease flare requiring treatment or new organ involvement and ESSDAI changes until flare or end of follow-up.

The secondary outcome of this study was to evaluate if the number of lymphocytic foci and the lymphoid composition of the MSGB show an association with the clinical characteristics and severity of Sjögren’s syndrome at diagnosis.

Severe disease flare was defined as new major organ damage, such as lymphoma, arthritis, nervous system, and renal or pulmonary affection, or a flare requiring moderate to high doses of steroids or add-on DMARD. The ESSDAI score was evaluated for every visit until the end of follow-up or until severe disease flare. Moreover, meaningful improvement of the ESSDAI was collected if there was a decrease of 3 points from the baseline compared to the baseline visit (15, 16). We defined the study’s outcomes to encompass different clinical scenarios: severe disease flare and a clinically meaningful ESSDAI improvement, which may include fluctuation without treatment.

### 2.4 MSGB and immunofluorescence staining

In our clinical practice, a plastic surgeon obtained four fragments of minor salivary glands, each with a diameter of 2 mm. The immunology department was responsible for the immunofluorescence staining of the glands. The entire minor salivary gland piece was sequentially sliced into 7-micron sections, and the biopsy was completely devastated. For every 10 sections, 1 was stained with methylene blue and observed under the microscope. In the presence of infiltrates, the slide was prepared for further staining with antibodies. If there was no infiltrate, the cutting process continued until an infiltrate was identified. For each sample, two slides from two different points of the infiltrate were processed. The numbers reported in the manuscript correspond to the largest infiltrate observed in the total of the three fragments. The cell numbers were manually counted in the largest infiltrate within one of the glands. If there were any variations or differences in the cell composition among the infiltrates, these were described independently.

Both cellular infiltrate and its lymphoid composition were assessed by immunofluorescence staining of sequential sections with monoclonal antibodies recognizing CD45, CD3, CD4, CD8, CD20, HLA-DR, kappa, and lambda immunoglobulin chains.

**Figure 1:**
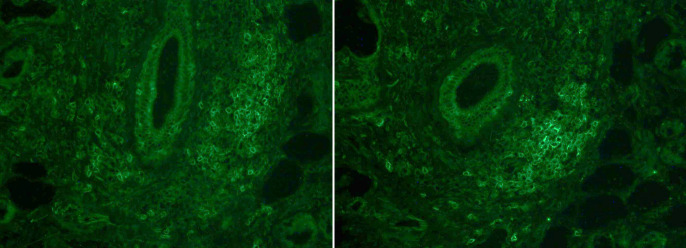
Immunofluorescence staining with anti-CD45 (left) and anti-CD20 (right).

Firstly, MSGB was embedded in O.C.T. Compounds (Sakura Finetek USA), frozen in liquid nitrogen, and cross-sections of 7 µm thickness were obtained (**Figure 1**). MSGB sections were fixed with acetone (Panreac Applichem, Barcelona, Spain) and incubated with the following primary antibodies: anti-CD45 for leukocyte identification (clone 2B11 and PD7/26), anti-CD3 for T lymphocytes (clone F7.2.38), anti-CD4 for T-helper lymphocytes (clone 4B12), anti-CD8 for cytotoxic T lymphocytes (clone C8/144B), anti-CD20 for B lymphocytes (clone L2), anti-HLA-DR for detecting MHC class II expression (clone TAL.1B5), and polyclonal anti-kappa and anti-lambda light chains for detecting immunoglobulins (DAKO, Glostrup, Denmark). After washing with PBS (Immuno concepts, Sacramento, California), sections were incubated with polyclonal rabbit anti-mouse immunoglobulins/FITC (DAKO, Glostrup, Denmark). All sections were randomly analyzed by two expert observers and blinded to clinical and molecular data. Each sample was independently evaluated, and any discrepancies were resolved by agreement.

A biopsy was considered lymphocytic foci positive when displaying at least one focus of lymphocyte infiltration with more than 50 cells within normal minor salivary gland tissue. Cellular infiltration that did not meet this criterion was classified as negative. A complete description of the characteristics of the lymphoid composition was performed.

### 2.5 Statistical analysis

We carried out a crude analysis using Student’s t-test, the Fisher–Pitman test was used to compare quantitative variables, and the Chi² test was employed to compare dichotomous variables according to outcomes of interest. Analysis was not carried out for variables with a small number of cases where assumptions were not met. The Shapiro–Wilk test and a box plot were used to check the distribution of the variables. For variables related to the MSGB findings, if log-normal distribution was observed, logarithmic transformation was carried out previously to apply regression or correlation tests.

For the primary outcome of the study, Cox regression was carried out to evaluate the risk of severe disease flare according to the number of lymphocytic foci and their lymphoid composition. Linear regression was used to evaluate the association between the ESSDAI changes and MSGB findings. Pearson and Kendall’s Tau correlation were calculated to evaluate the strength of association.

For the secondary outcome of the study, linear regression was used to evaluate the association between the ESSDAI at diagnosis, clinical variables, and MSGB findings.

In the previous statistical plan established, the presence of other systemic autoimmune diseases, years since first symptoms, steroid treatment, DMARDs, sex, and age were selected as clinically relevant variables for adjustment if meaningful association was found from the crude analysis. A two-sided 95% confidence interval was calculated, and a p-value <0.05 was considered significant. Data analysis was performed using Stata 16.0.

## 3 Results

### 3.1 Baseline population characteristics

A total of 139 individuals met the eligibility criteria, of whom 81 fulfilled the inclusion criteria. Ultimately, 78 patients were included after excluding 3 individuals due to insufficient salivary gland tissue samples. There was no loss during the 3-year follow-up period. The baseline characteristics and comparison of groups according to the main outcomes are shown in **Table 1**. In all, 13 patients tested positive for Ro/La antibodies, with 3 showing positivity for all three antibodies (Ro52/Ro62/La). Additionally, 6 patients were positive for both Ro52 and Ro60, while 4 patients tested positive for either Ro52 or Ro60 alone. **Supplementary Figure s 1–9** summarizes the distribution of the cellular infiltration of the lymphocytic foci.

**Table 1:** Baseline characteristics and comparison of main outcomes.

Variable	Progression (n=16)	Stable disease (n=62)	p	ESSDAI improvement (n=8)	No ESSDAI improvement (n=70)	p	Total (n=78)
Male sex, (n, %)	4 (25)	4 (6.45)	0.03	1 (12.5)	7 (10)	0.83	8 (10.26)
Age, mean (SD)	56.35 (14.60)	61.73 (12.05)	0.13	64.67 (5.89)	60.17 (13.21)	0.35	60.63 (12.70)
Years since disease symptoms, p50 (IQR)	3.5 (2)	4.5 (10)	0.30	3 (2.5)	4 (9)	0.12	4 (9)
Baseline ESSDAI, mean (SD) Inactive to low disease, (n, %) Moderate or higher activity, (n, %)	3.81 (3.97) 8 (50) 8 (50)	1.06 (1.85) 56 (90.32) 6 (9.68)	0.01	6.75 (1.98) 0 8 (100)	1.04 (2.01) 64 (91.43) 6 (8.57)	0.00	1.63 (2.65) 64 (82.05) 14 (17.95)
Follow-up ESSDAI, mean (SD)	3.5 (4.49)	0.53 (1.16)	0.00	0.88 (0.83)	1.17(2.66)	0.29	1.14 (2.54)
Other autoimmune disease, (n, %)	4 (25)	9 (14.52)	—	2 (50)	11 (15.71)	—	13 (16.67)
Lymphoma, (n, %)	3 (18.75)	2 (3.23)	—	0	5 (7.14)	—	5 (6.41)
ANA titers, (n, %) <1/80 1/80 1/160 1/320 ≥1/640	5 (31.25) 3 (18.75) 1 (6.25) 0 7 (43)	14 (22.58) 15 (24.19) 14 (22.58) 7 (11.29) 12 (19.36)	0.52	3 (37.5) 1 (12.5) 1 (12.5) 0 3 (37.5)	16 (22.86) 17 (24.29) 14 (20) 7 (10) 16 (22.86)	0.87	19 (24.36) 18 (23.08) 15 (19.23) 7 (8.97) 19 (24.36)
Ro/La antibody, (n, %)	7 (43.75)	6 (9.68)	0.07	3 (37.5)	9 (12.86)	0.23	13 (16.67)
Rheumatoid Factor, (n, %)	4 (25)	10 (16.12)	0.50	2 (25)	12 (17.14)	0.66	14 (17.95)
Normal gammaglobulinemia, (n, %)Hypergammaglobulinemia, (n, %) Hypogammaglobulinemia, (n, %)	12 (75) 0 4 (25)	47 (75.81) 4 (6.45) 3 (4.84)	0.08	6 (75) 0 2 (25)	61 (87.14) 4 (5.71) 5 (7.14)	0.17	67 (85.90) 4 (5.13) 7 (8.97)
Hypocomplementemia, (n, %)	6 (37.5)	13 (20.97)	0.34	4 (50)	15 (21.43)	–	19 (23.36)
History of steroid treatment, (n, %)	2 (12.5)	9 (14.52)	–	1 (12.5)	10 (14.29)	–	11 (14.10)
History of DMARD use, (n, %)	2 (12.5)	2 (3.23)	–	0	4 (5.71)	–	4 (5.13)
Steroid treatment at diagnosis, (n, %)	6 (37.5)	0	–	2 (25)	4 (5.71)	–	6 (7.69)
DMARD use at diagnosis, (n, %)	6 (37.5)	2 (3.23)	–	1 (12.5)	7 (10)	–	8 (10.26)

Comparison was not carried out in a small number of cases.

When comparing patients who experienced severe disease flare with those who maintained stable disease, a higher prevalence of male sex, history of lymphoma, and treatment with DMARDs and steroids was observed. When comparing patients who demonstrated significant ESSDAI improvement at the end of the 3-year follow-up period, a higher prevalence of steroid use at diagnosis and a higher baseline ESSDAI were observed, as one would expect.

Within our cohort, it is noteworthy that nearly 44.59% of patients demonstrated ANA titers lower than 1/160, and 87.38% of patients were negative for Ro and La antibodies. The lymphoid infiltration in the biopsy was similar between patients with low and high ANA titers and between Ro/La antibody-positive and -negative groups. No significant differences were observed when comparing the lymphoid composition of the biopsy between groups.

### 3.2 Association with disease severity at diagnosis and severe disease flare

Among patients who exhibited severe disease flare during the 3-year follow-up period, higher total lymphocyte counts, along with elevated T-cell, T CD4+, and T CD8+ cell counts, were observed in the lymphocytic foci when compared to patients with stable disease. No significant differences were noted in the lymphoid composition of the lymphocytic foci between patients who demonstrated ESSDAI improvement and those who did not. **Table 2** provides a summary of the key findings concerning the lymphoid composition of the lymphocytic foci of the MSGB, along with a comparison based on the main outcomes.

**Table 2:** Lymphoid composition of the lymphocytic foci of the MSGB and comparison of main outcomes.

Variable	Progression (n=16)	Stable disease (n=62)	p	ESSDAI improvement (n=8)	No ESSDAI improvement (n=70)	p	Total (n=78)
Number of lymphocytic foci, (n, %) 0 1 2 ≥3	1.5 1 (6.25) 7 (43.75) 2 (12.5) 6 (37.5)	1 6 (9.68) 33 (53.23) 13 (20.97) 10 (16.13)	0.10	1 1 (12.5) 4 (50) 1 (12.5) 2 (25)	1 6 (8.57) 36 (51.43) 14 (20) 14 (20)	0.94	1.53 (0.95) 7 (8.97) 40 (51.28) 15 (19.23) 16 (20.51)
Total number of lymphocytes, mean (SD)	191.56 (256.17)	121.04 (112.52)	0.12	100.63 (101.26)	139.5 (158.68)	0.58	85 (120)
Number of T cells, mean (SD)	115 (157.05)	58.95 (38.77)	0.03	45.63 (40.57)	73.29 (83.80)	0.29	55 (55)
Number of T CD4 cells, mean (SD)	82.11 (103.20)	41.4 (25.14)	0.03	32.92 (25.16)	51.26 (54.78)	0.33	35 (36.67)
Number of T CD8 cells, mean (SD)	49.32 (67.12)	24.69 (17.67)	0.04	27.92 (21.79)	29.86 (35.88)	0.85	18.33 (16.25)
Number of B cells, mean (SD)	76.56 (132.71)	62.10 (84.06)	0.57	55 (76.25)	66.21 (72.25)	0.86	30 (90)
% T cells/Total lymphocytes, mean (SD)	69.48 (29.91)	63.40 (27.98)	0.46	0.53 (0.37)	0.66 (0.27)	0.26	0.65 (0.28)
% B cells/Total lymphocytes, mean (SD)	30.52 (29.91)	36.60 (27.98)	0.46	0.47 (0.37)	0.34 (0.27)	0.26	0.35 (0.28)
% T CD4 cells/Total T cells, mean (SD)	64.29 (11.98)	62.64 (15.29)	0.73	0.56 (0.18)	0.64 (0.14)	0.19	0.63 (0.15)
% T CD8 cells/Total T cells, mean (SD)	35.71 (12.0)	37.36 (15.29)	0.77	0.44 (0.18)	0.36 (0.14)	0.25	0.37 (0.15)
Presence of isolated infiltrate without lymphocytic foci, (n, %)^+^	3 (18.75)	11 (17.74)	—	1 (12.5)	13 (18.57)	—	14 (17.95)

Comparison was not carried out in a small number of cases (<5).

Cox regression was employed to examine the prognostic value of lymphoid composition of the lymphocytic foci and severe disease flare. Among the studied risk factors, only the number of T cells exhibited statistical significance for severe disease flare, displaying a hazard ratio (HR) of 1.01 (95% CI 1.00-1.01); this effect, however, was clinically insignificant. Given the lognormal distribution of variables related to total cell counts (**Figure 1**), a logarithmic transformation was applied to these variables. The HR for the logarithmically transformed T-cell count was 1.96 (95% CI 0.91 – 4.21), indicating a moderate risk effect that approached statistical significance. No discernible prognostic correlation was identified for the count of T CD4+ cells, T CD8+ cells, or the total number of lymphocytes, consistent with the results of the initial analysis. Furthermore, no associations were evident in relation to the proportion of each cell subtype.

Linear regression was employed to analyze differences in ESSDAI scores between follow-up and baseline visits. A higher difference signifies an enhancement, while a lower difference indicates deterioration in the ESSDAI. Variations in ESSDAI scores showed positive associations with total lymphocyte count, T- and B-cell numbers, and the logarithm of T CD4+ cells (suggesting a potential exponential relationship for the latter) within the lymphocytic foci. **Table 3** summarizes the results of the regression analysis for the main outcomes. Although the strength of association was assessed through correlation analysis, no statistically or clinically significant relationships were observed.

**Table 3:** Evaluation of the lymphoid composition of the lymphocytic foci using Cox regression for severe disease flare and linear regression for ESSDAI differences.

Variable	HR (95% CI) for severe disease flare	p	β (95% CI) for ESSDAI difference from baseline	p
Number of lymphocytic foci, (n, %) 0 1 2 ≥3	1.57 (0.92 - 2.69) 1.22 (0.15 - 10.12) 1.02 (0.09 - 11.24) 2.59 (0.29 - 23.32) 14.20 (0.84 - 240.23)	0.10 0.89 0.99 0.40 0.07	0.59 (-0.00 - 1.18) -0.24 (-2.28 - 1.80) 0.31 (-1.97 - 2.59) 1.45 (-0.83 - 3.73) 0.71 (-4.6 - 6.04)	0.05 0.82 0.78 0.21 0.79
Total number of lymphocytes, mean (SD)	1.00 (1.00 - 1.01)	0.17	0.01 (0.00 - 0.01)	0.00
Log total lymphocytes, mean (SD)	1.13 (0.61 - 2.08)	0.70	0.52 (-0.14 - 1.18)	0.12
Number of T cells, mean (SD)	1.01 (1.00 - 1.01)	0.01	0.01 (0.00 - 0.15)	0.03
Log T cells, mean (SD)	1.96 (0.91 - 4.21)	0.09	0.70 (-0.11 - 1.51)	0.09
Number of T CD4 cells, mean (SD)	0.87 (0.53 - 1.43)	0.58	0.26 (-0.30 - 0.82)	0.35
Log T CD4+ cells, mean (SD)	1.79 (0.82 - 3.89)	0.14	0.91 (0.09 - 1.73)	0.03
Number of T CD8 cells, mean (SD)	0.74 (0.30 - 1.84)	0.50	-0.06 (-0.96 - 0.83)	0.89
Log T CD8+ cells, mean (SD)	1.68 (0.82 - 3.42)	0.16	0.61 (-0.20 - 1.42)	0.14
Number of B cells, mean (SD)	1.00 (1.0 - 1.01)	0.80	0.01 (0.00 - 0.015)	0.00
Log B cells, mean (SD)	0.71 (0.39 - 1.31)	0.27	0.71 (0.07 - 1.34)	0.03
% T cells/Total lymphocytes, mean (SD)	2.51 (0.33 - 18.91)	0.37	0.04 (-2.09 - 2.16)	0.97
% B cells/Total lymphocytes, mean (SD)	0.40 (0.53 - 2.99)	0.37	-0.04 (-2.16 - 2.09)	0.97
% T CD4 cells/Total T cells, mean (SD)	1.01 (0.20 - 49.98)	1.00	1.31 (-2.65 - 5.27)	0.51
% T CD8 cells/Total T cells, mean (SD)	0.99 (0.02 - 49.38)	1.00	-1.31 (-5.27 - 2.65)	0.51
Presence of isolated infiltrate without lymphocytic foci, (n, %) ^+^	1.42 (0.39 - 5.11)	0.61	0.07 (-1.42 - 1.56)	0.92

### 3.3 Association with clinical and serological manifestations

The relationship between the clinical characteristics and lymphoid composition of the lymphocytic foci was explored using group comparisons, correlation analysis, and regression analysis. Seropositive patients displayed a higher number of T CD4+ cells, with a mean of 100.83 (SD 122.71), in contrast to seronegative patients with a mean of 43.49 (SD 36.81); this difference was statistically significant (p=0.04). Correlation analysis revealed that age exhibited a negative correlation with the number of lymphocytic foci with a tau-b score of -0.17 (p=0.05) and a negative correlation with the number of T cells with a tau-b score of -0.15 (p=0.05). Antinuclear antibody (ANA) titers demonstrated positive correlations with the total number of lymphocytes with a tau-b score of 0.1768 (p=0.04), a positive correlation with the number of T cells with a tau-b score of 0.22 (p=0.01), and a positive correlation with the number of T CD8+ cells with a tau-b score of 0.19 (p=0.03). However, the linear regression analysis did not reveal any significant association between the clinical variables and lymphoid composition of the lymphocytic foci including serological variables, DMARD, or steroid treatment. No further multiple analysis was performed for this reason, but confounder analysis revealed that these variables were not found to be confounders or modifiers of effect.

## 4 Discussion

Our study found significant associations between clinical factors and severe disease flare, with male sex, lymphoma history, and DMARDs/steroid use being more common in progressing patients. Increased lymphocyte and T-cell counts within lymphocytic foci suggest immune cell involvement in exacerbations. The total T-cell count within lymphocytic foci was significantly associated with severe disease flare (HR 1.01) after logarithmic transformation. The subsequent Cox regression showed a substantial risk between T-cell count within lymphocytic foci and severe disease flare (HR 1.96, approaching significance).

Regarding ESSDAI score changes and lymphoid composition of lymphocytic foci, there was a positive link between ESSDAI improvement and T CD4+ and B-cell counts post-logarithmic transformation, with weak associations with T cells and overall lymphocytes within lymphocytic foci. No significant relationships emerged with cell subtype proportion or cellular infiltrates lacking germinal centers.

Concerning the correlation between clinical and serological variables and the MSGB’s lymphoid composition of lymphocytic foci, seropositive patients exhibited elevated T CD4+ cell counts. Age was inversely correlated with the number of lymphocytic foci and its T-cell counts. ANA titers positively correlated with total lymphocyte, T-cell, and T CD8+ cell counts. It is important to note that these correlations are not sufficient for making predictions or definitive conclusions. No significant relationships were observed with the duration of disease symptoms, disease biological activity, or steroid/DMARD treatment.

Our study population exhibited characteristics consistent with prior research, confirming a higher prevalence of male sex, lymphoma history, and the use of steroids or DMARDs in patients with poorer prognosis. Our study included incident cases of Sjögren’s syndrome, accompanied by a low prevalence of treatment history that could potentially impact the results of the MSGB. Despite a relatively short median time from disease symptom onset, our study population encompassed patients ranging from less than 1 year to 30 years after initial manifestation. Severe disease flare was considered only if new major organ damage was observed or if moderate to high doses of steroid or DMARD treatment were required.

The focus score, based on the histopathologic criteria established by Greenspan et al. in 1974 (10), has been known to potentially overestimate cell infiltrate extent (11, 12) with poorer interobserver reliability than GC-like structure assessment (13). In routine hematoxylin and eosin (H&E) staining, most leukocytes generally appear quite similar in terms of their staining characteristics. While this staining method is excellent for visualizing tissue architecture, it may be challenging to distinguish lymphocytes from other leukocyte subtypes. Immunofluorescence staining, allowing for the visualization and quantification of specific cellular components and lymphoid composition within salivary gland tissue, has the potential to significantly improve diagnostic accuracy while reducing interobserver discrepancies. As demonstrated in a systematic review, the percentage of MSGBs with a focus score exceeding 1 increased from 63% to 83% when immunohistochemical staining was used to highlight small clusters of lymphocytes that might otherwise go unnoticed when intermingled with salivary ducts or acini (14).

Thus, the use of immunofluorescence to evaluate cell subtypes aimed to enhance diagnostic accuracy. This approach elucidates why our study population included patients with infiltrates exhibiting a high lymphocyte count, accompanied by the presence of lymphocytic foci in all enrolled patients.

At our institution, MSGB evaluation is conducted within the immunology department as part of standard clinical practice. This evaluation process is meticulous and involves two independent evaluators supervised by an expert immunologist. Sequential sections of the biopsy sample are completely devastated, encompassing the identification of lymphocytic foci and a detailed description of lymphoid composition. To ensure the highest accuracy, only lymphocyte aggregates containing over 50 cells are considered as foci indicative of Sjögren’s syndrome to minimize the risk of false positive diagnoses. In our study, a unique characteristic of the enrolled patient population was the presence of at least one lymphocytic focus in all patients meeting this criterion. Biopsies with cellular infiltrates lacking lymphocytic foci composed of fewer than 50 lymphocytes were considered inconclusive for Sjögren’s syndrome diagnosis. The lymphocytic foci described in our study resemble the germinal center (GC)-like structures defined by Costa et al. (17). However, further investigations by Carubbi et al. (18), Haacke et al. (19), and Kroese et al. (11) emphasize the importance of additional staining techniques, including CD21, CD3, CD20, and BCL-6, for a more precise characterization of germinal centers. Immunofluorescence serves to distinguish these lymphocytic foci that resemble GC-like structures from the diffuse lymphocytic infiltration of the gland. Interestingly, according to a systematic review, previous studies evaluating the MSGB had reported a low prevalence of germinal center-like structures, ranging from 18% to 59% (20).

Our study did not reveal any prognostic or clinical associations pertaining to the number of lymphocytic foci over the 3-year follow-up period. The infrequent instances of lymphoma cases in our study can be attributed to the relatively brief follow-up period, which was deliberately chosen to address the primary aim of evaluating the practical utility of the MSGB findings for short-term decision-making. Nonetheless, the prognostic value of the MSGB concerning its response to lymphoma treatment remains inconclusive (17). Our results show that the higher number of T cells in the lymphocytic infiltrates of lymphocytic foci may have an exponential relationship with clinically relevant severe disease flare in 3 years. Possibly, patients with a higher number of T cells in the lymphocytic infiltrates of lymphocytic foci may have two-fold risk of severe disease flare and are correlated with younger age. Moreover, in our population, the number of B cells and T CD4+ cells in the lymphocytic infiltrates of lymphocytic foci showed a weak but positive relation with ESSDAI improvement during follow-up. This result is different from some previous research where B-cell predominance was observed for longer disease evolution, extraglandular manifestations, and lymphoma (17, 21). The results also differ from studies that conclude that the number of B cells and T cells was not related to any clinical variables of severity, as in Carubbi et al. and Christoudoulou et al. (18, 22).

The natural history of minor salivary gland alterations in SS patients remains unclear. Until now, the FS and fibrosis were believed to increase over time and with severe disease flare (23, 24). However, in a recent study of serial MSGBs, neither the infiltration grade nor the FS values changed significantly, although significant severe disease flare occurred (24). Neither did significant changes develop for lymphocytic foci, fibrosis, or adiposis. This would indicate that SS lesions are slowly progressing alterations.

This study’s notable strength lies in its pioneering approach, being the first to examine the prognostic significance of the lymphoid composition within the lymphocytic foci of the MSGB in a population where all individuals exhibit lymphocytic foci. Additionally, the study’s robustness stems from its deliberate selection of severe disease flare as the primary outcome, driven by its clinical utility and relevance. The relatively brief follow-up duration was strategically chosen to capture the biopsy’s potential for guiding short-term decision-making, further enhancing the study’s practical applicability.

The primary limitation of this study was the sample size. It was conducted as a pilot study with consecutive patient inclusion over a 1-year recruitment period. As a result, it was not feasible to incorporate some statistical analysis due to the limited number of cases as indicated in the methods. Moreover, only univariate regression was possible. These limitations were addressed by employing non-parametric tests, which offer a robust method for comparing distributions without necessitating specific distributional assumptions. We acknowledge another limitation stemming from its monocentric nature. Our institution, a prominent referral tertiary hospital catering to a population of 450,000 residents, serves as an expertise unit and regional referral center in Catalonia. Despite this specialized role, the single-center design of our study might raise questions about the broader applicability of our findings.

However, it is important to note that this limitation is mitigated by several factors. Firstly, our hospital’s role as a comprehensive referral center ensures that a diverse range of patients seeking evaluation for Sjögren’s syndrome, and subsequently undergoing MSGB, were included in our study. This, in turn, contributes to the representation of a wider spectrum of cases. Moreover, the standardized protocol followed for MSGB examination across all patients ensures consistency in the procedure, minimizing variability in this critical aspect of our study.

In light of these considerations, while the monocentric nature of our study could potentially influence the external generalizability of our findings, the comprehensive scope of our institution’s services and the standardized procedures employed help mitigate this limitation to some extent.

In conclusion, our study has revealed increased counts of lymphocytes, T cells, T CD4+, and T CD8+ cells in the lymphocytic foci of the MSGB in patients with progressing disease, suggesting an active role for immune cells in exacerbations. Particularly, the total T-cell count exhibited a significant association with severe disease flare, indicating its potential as a prognostic marker. Our subsequent Cox regression analysis unveiled a substantial risk between T-cell count and severe disease flare with a HR of 1.96, though statistical power limitations might have hindered reaching full significance.

Analyzing the relationship between the ESSDAI score changes and lymphoid composition of the lymphocytic foci, our linear regression uncovered a positive link between ESSDAI improvement and post-logarithmic transformation T CD4+ and B-cell counts. While similar associations were found with T cells and overall lymphocytes, their coefficients were indicative of weak connections. Importantly, no significant relationships emerged regarding cell subtype proportions or the presence of isolated lymphocytic infiltrate without foci.

Future research endeavors involving multiple centers and bigger sample sizes could offer a broader perspective and further validate the implications of our study.

## Data availability statement

The raw data supporting the conclusions of this article will be made available by the authors, without undue reservation.

## Ethics statement

The studies involving humans were approved by IIB Research Institute Sant Pau. The studies were conducted in accordance with the local legislation and institutional requirements. The human samples used in this study were acquired from a by-product of routine care or industry. Written informed consent for participation was not required from the participants or the participants’ legal guardians/next of kin in accordance with the national legislation and institutional requirements.

## Author contributions

H-SP: Conceptualization, Data curation, Formal analysis, Funding acquisition, Investigation, Methodology, Project administration, Writing – original draft, Writing – review & editing. LM-M: Conceptualization, Data curation, Formal analysis, Investigation, Methodology, Supervision, Validation, Writing – original draft, Writing – review & editing. BM: Conceptualization, Data curation, Formal analysis, Investigation, Methodology, Project administration, Supervision, Writing – review & editing. IC: Conceptualization, Investigation, Supervision, Writing – review & editing. PM: Conceptualization, Methodology, Supervision, Writing – review & editing. HC-M: Data curation, Investigation, Formal analysis, Validation, Writing – review & editing. NH: Investigation, Supervision, Validation, Writing – review & editing. CD-T: Conceptualization, Formal analysis, Methodology, Supervision, Writing – review & editing. AL: Conceptualization, Supervision, Writing – review & editing. LS: Conceptualization, Data curation, Supervision, Validation, Writing – review & editing. JT: Conceptualization, Investigation, Methodology, Writing – review & editing. AM-R: Methodology, Supervision, Writing – review & editing. TF-L: Conceptualization, Data curation, Investigation, Supervision, Writing – review & editing. JC: Data curation, Investigation, Methodology, Writing – review & editing, Conceptualization. CJ: Conceptualization, Investigation, Methodology, Supervision, Writing – review & editing. HC: Conceptualization, Data curation, Formal analysis, Funding acquisition, Investigation, Methodology, Supervision, Validation, Writing – original draft, Writing – review & editing.
