# Supplementary material for: Prognostic significance of lymphocytic foci composition in minor salivary gland biopsies for severe disease flare and severity in Sjögren’s syndrome: a 3-year follow-up cohort study
Source: Front Immunol. 2024 Feb 26;15:1332924. doi: 10.3389/fimmu.2024.1332924 (PMC10925694; doi:10.3389/fimmu.2024.1332924)

**Figure 1. Distribution of cell count related variables of the MSGB.**


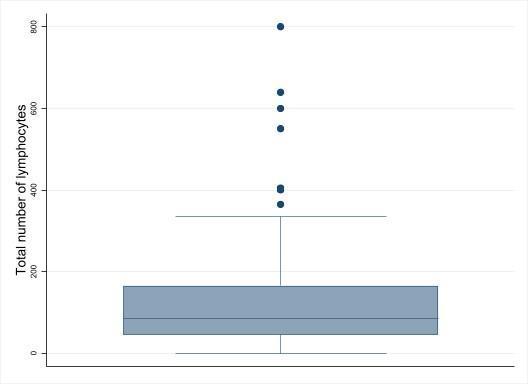

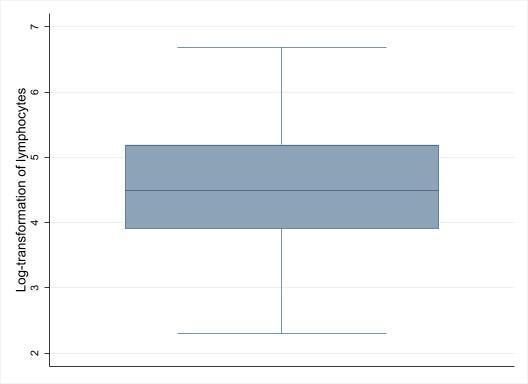

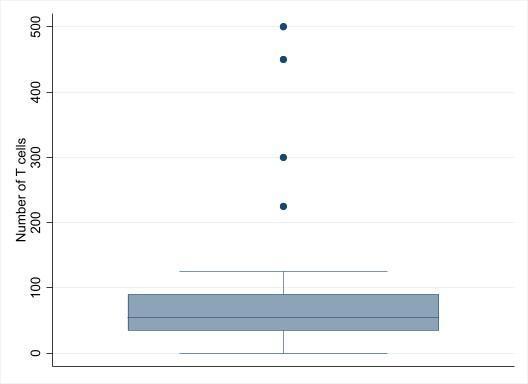

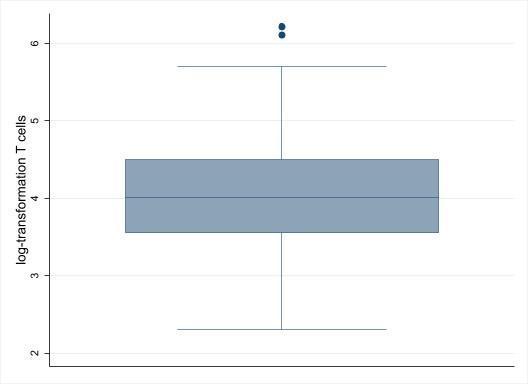

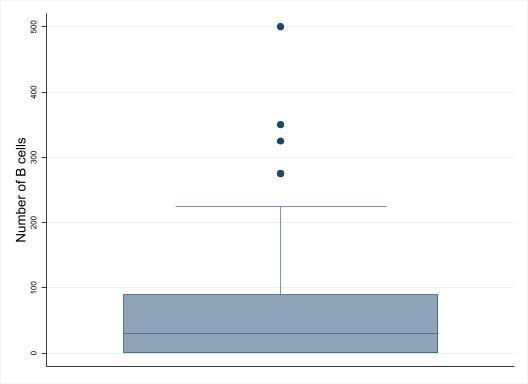

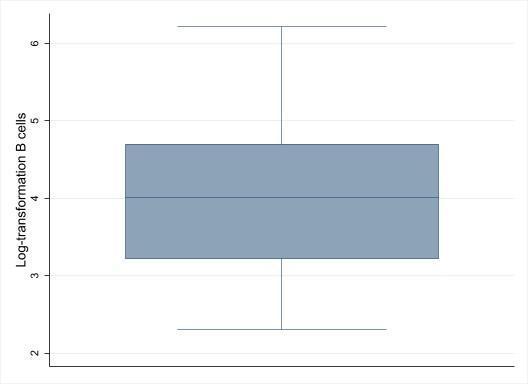


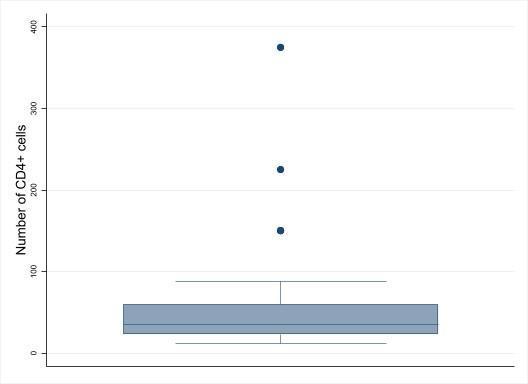

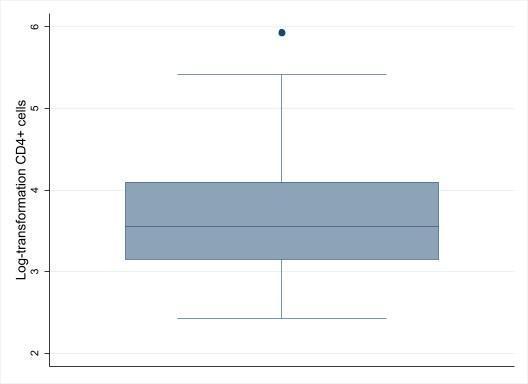


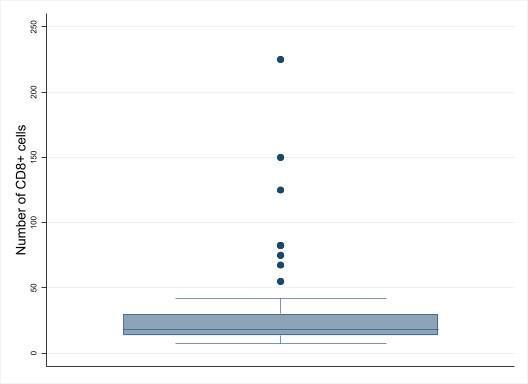

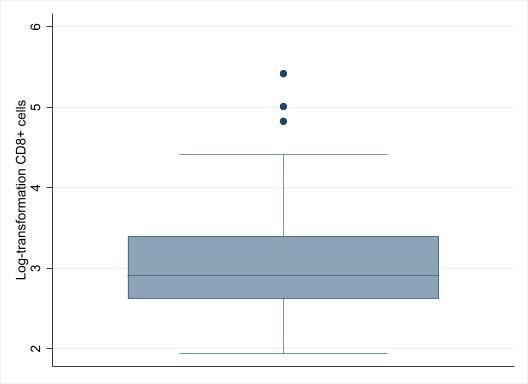


**Figure 2. Correlation between total number of lymphocytes, clinical and serological variables.**


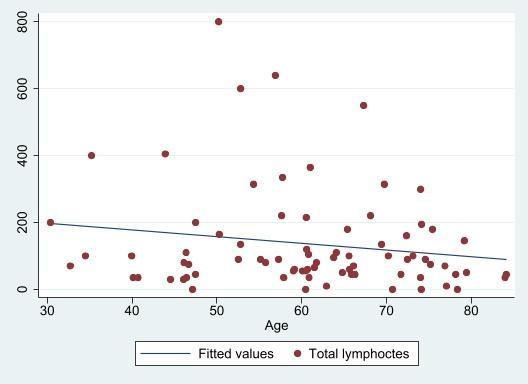

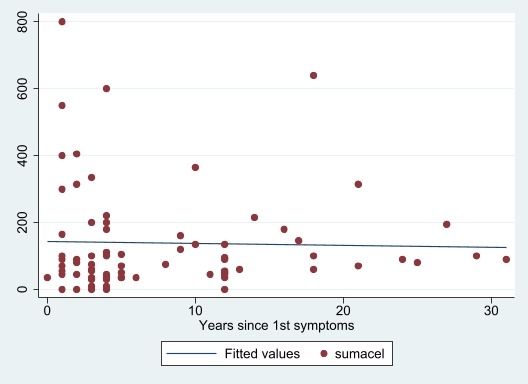

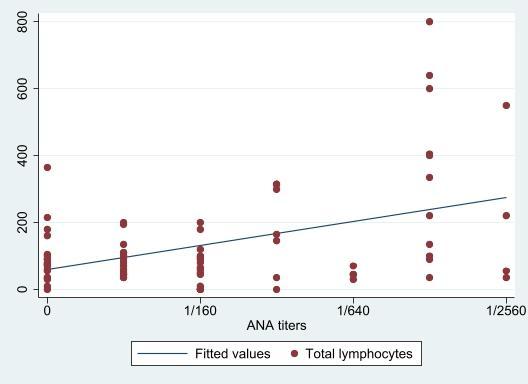

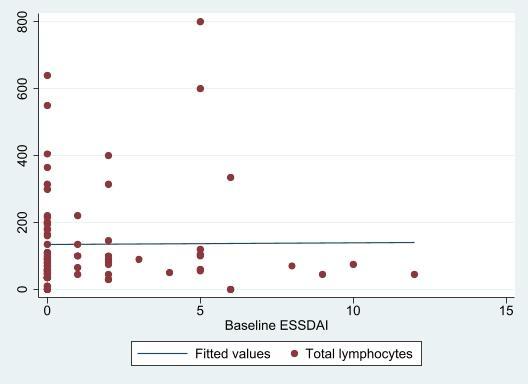

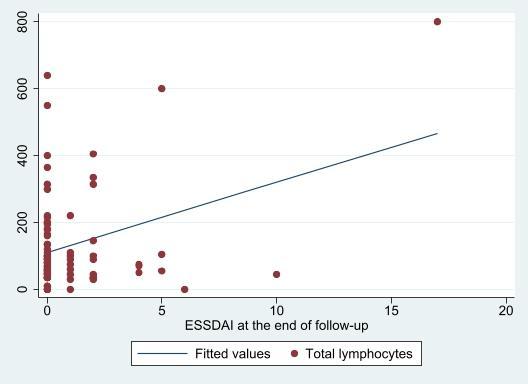


**Figure 3. Correlation between total number of T cells, clinical and serological variables.**


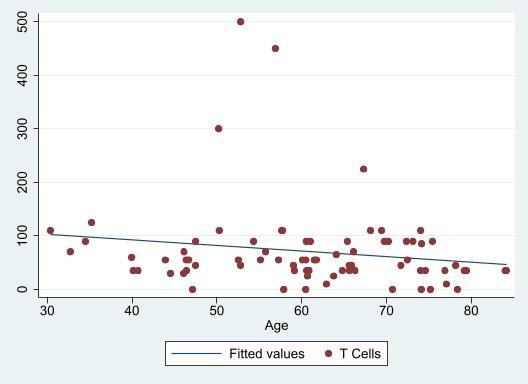

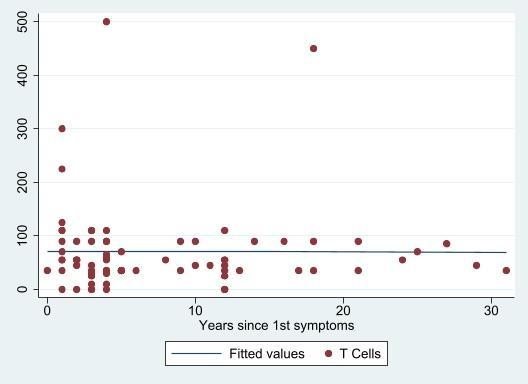


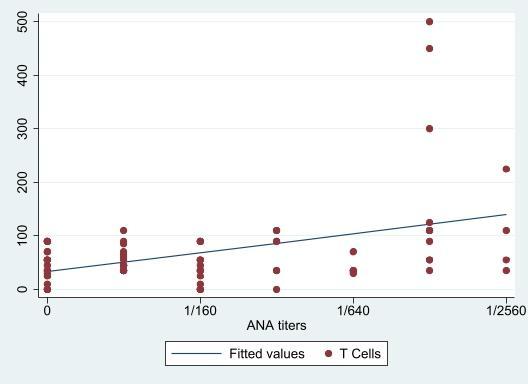

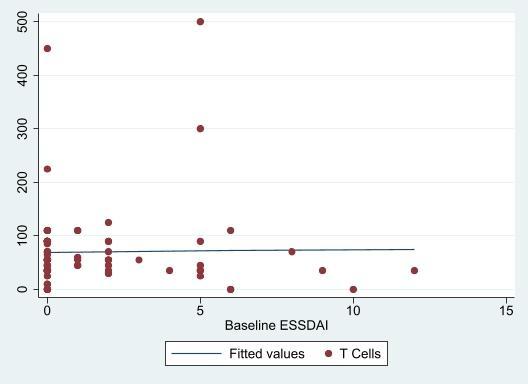


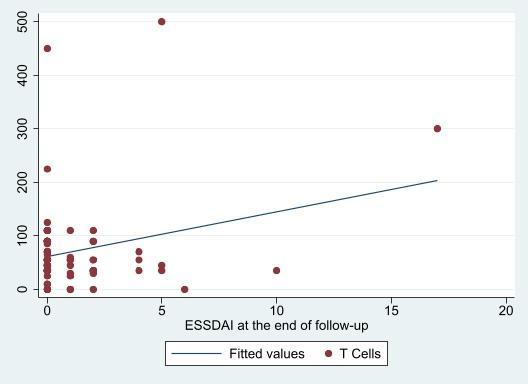


**Figure 4. Correlation between B Cells, clinical and serological variables.**


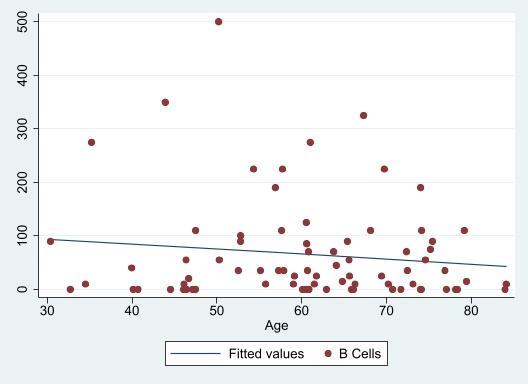

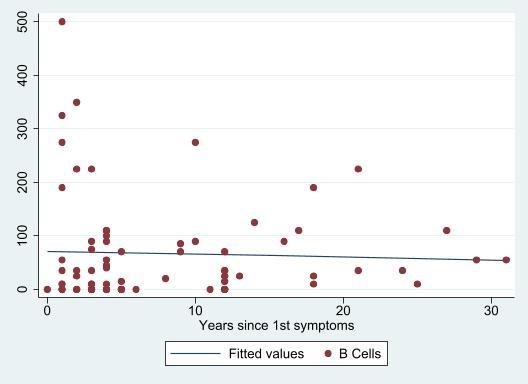


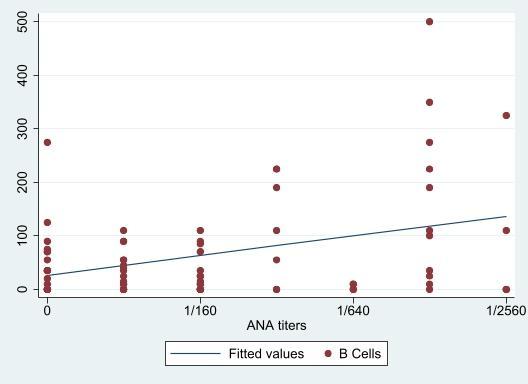

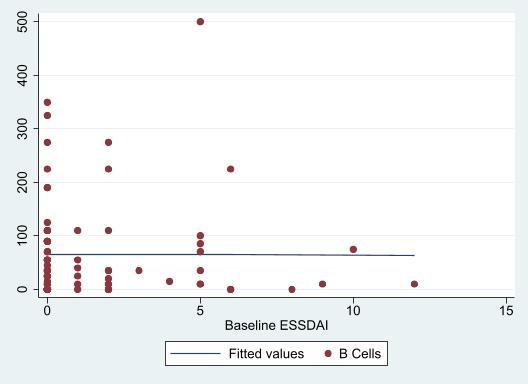


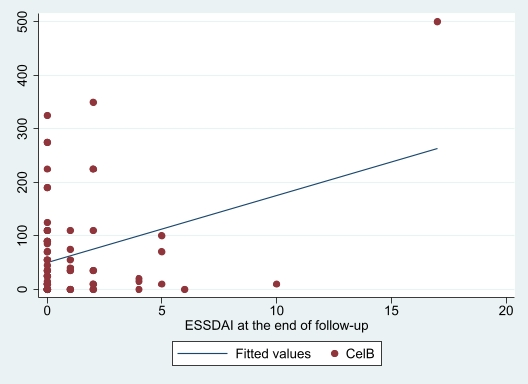


**Figure 5. Correlation between total number of CD4 Cells, clinical and serological variables.**


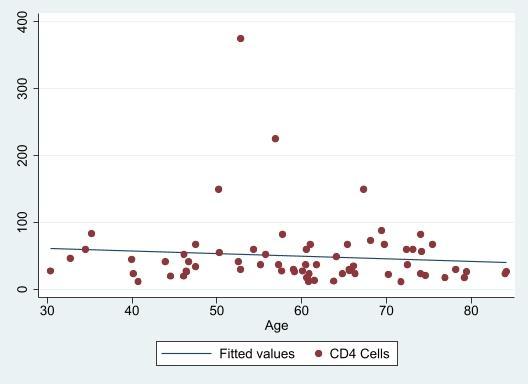

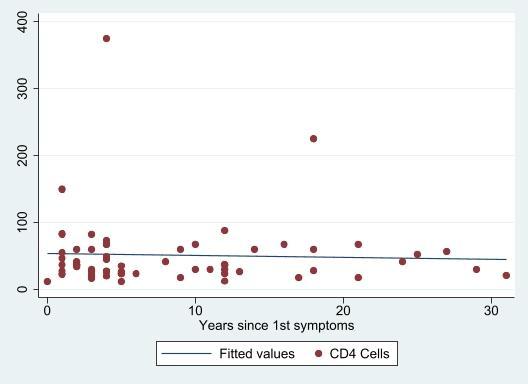

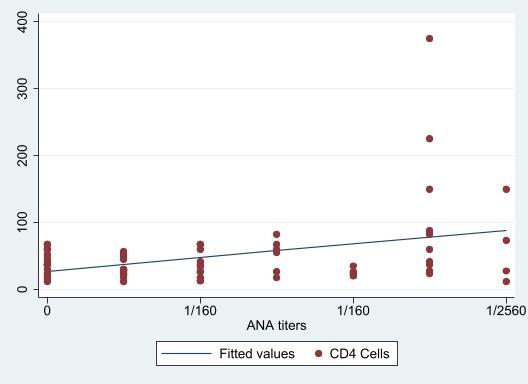

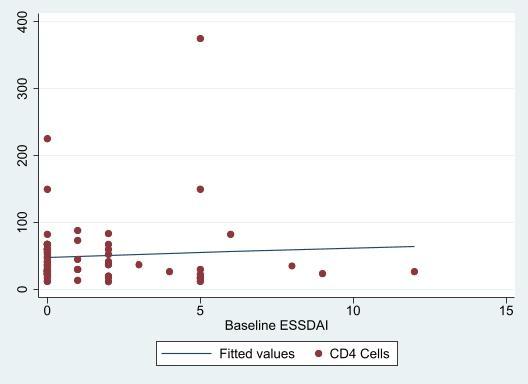


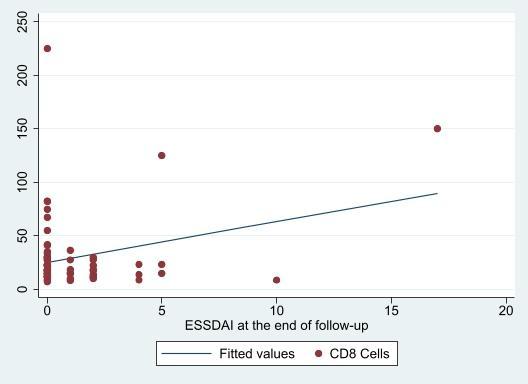


**Figure 6. Correlation between total CD8+ Cells, clinical and serological variables.**


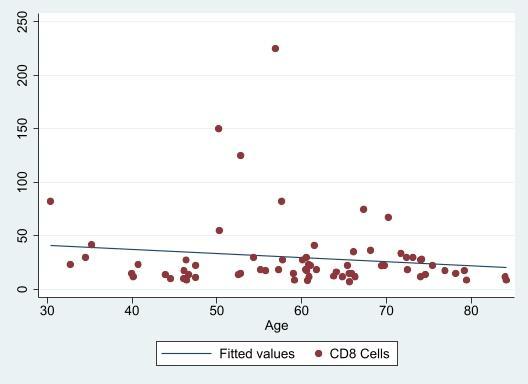

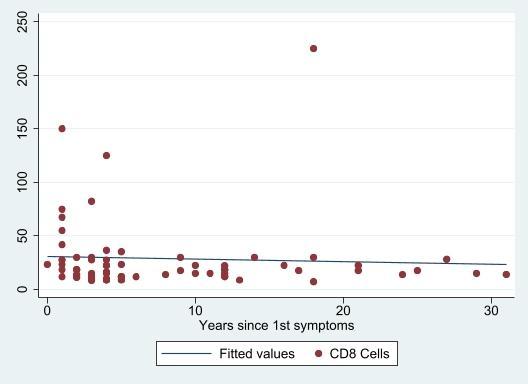


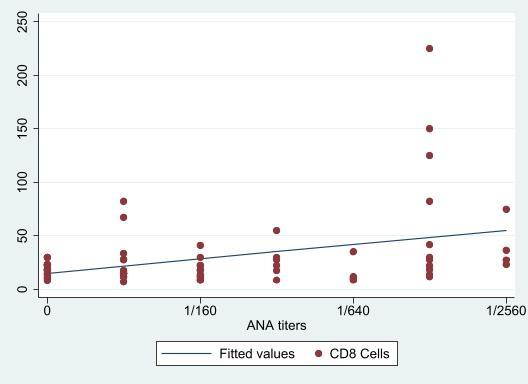

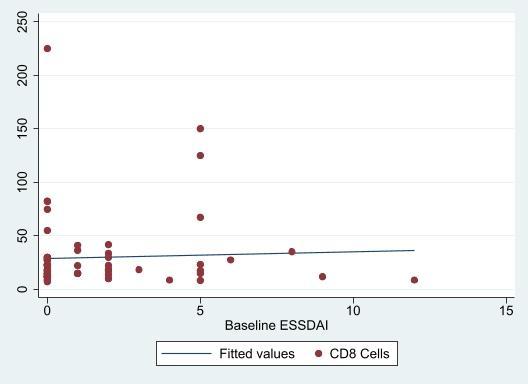


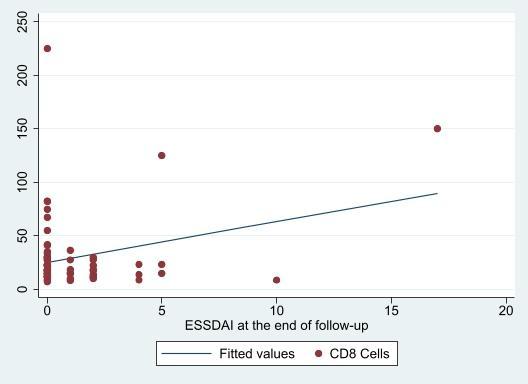


**Figure 7. Bar graphs of mean (SD) cell count for patients with ESSDAI improvement vs no improvement.**


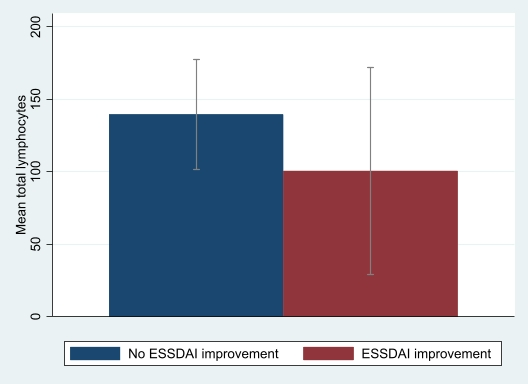

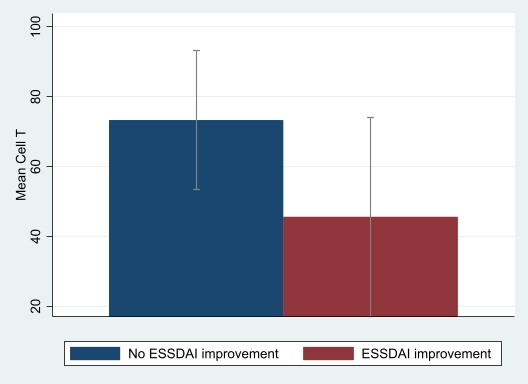

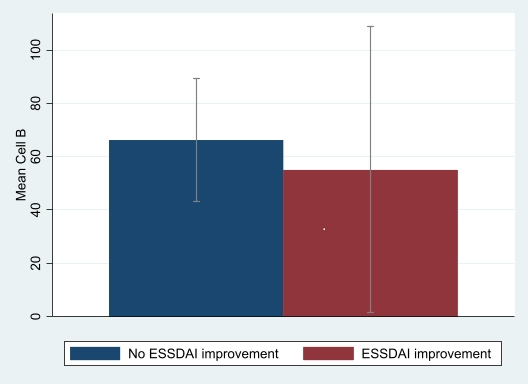

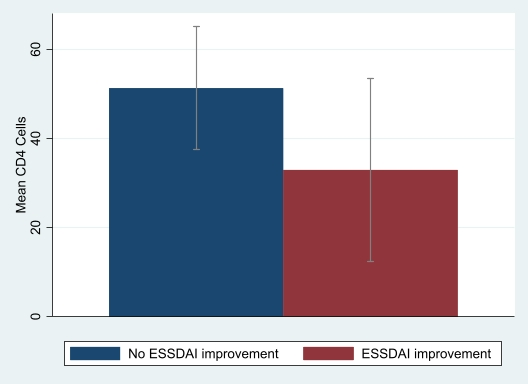


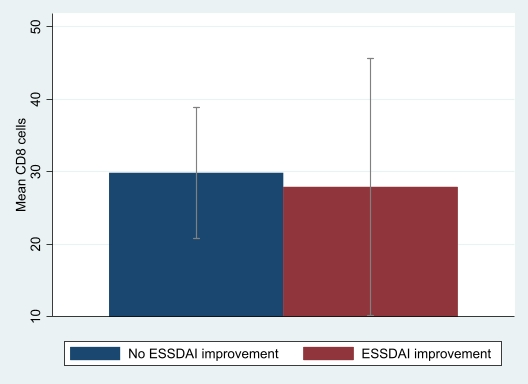


**Figure 8. Bar graphs of mean (SD) cell count for patients with lymphoma vs no lymphoma.**


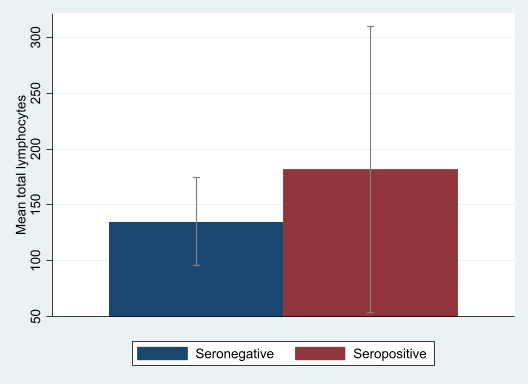

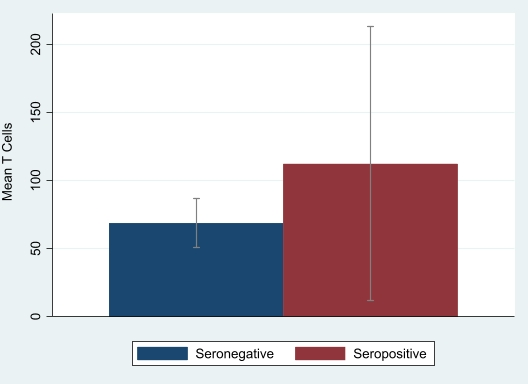

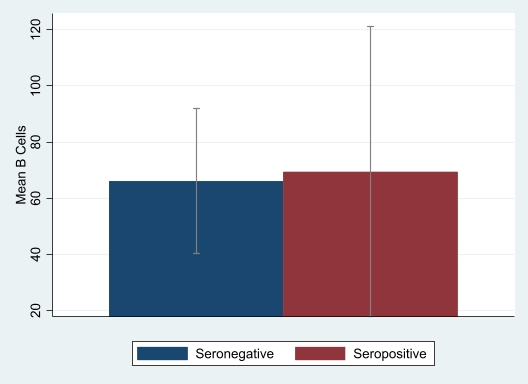

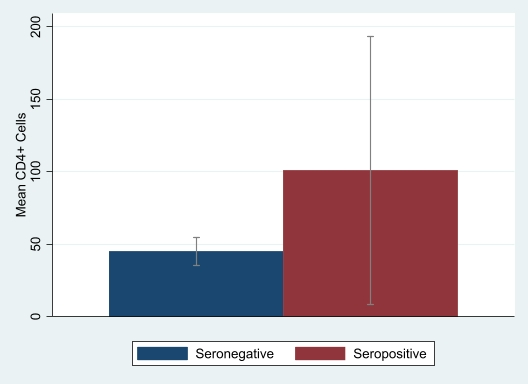

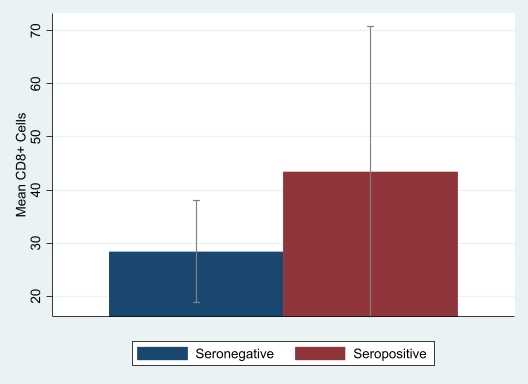


**Figure 9. Bar graphs of mean (SD) cell count for patients with seropositivity vs seronegativity.**


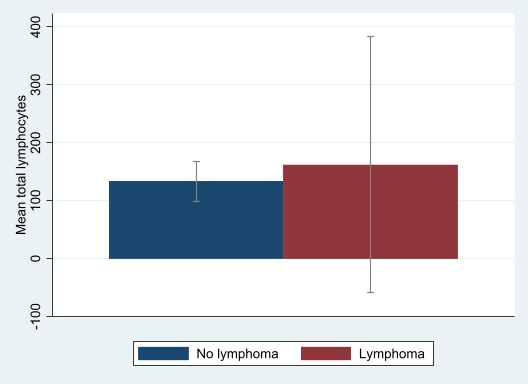

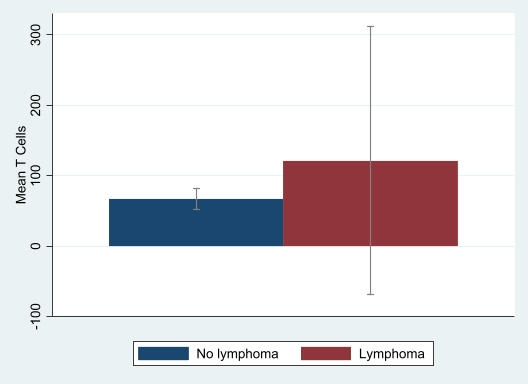

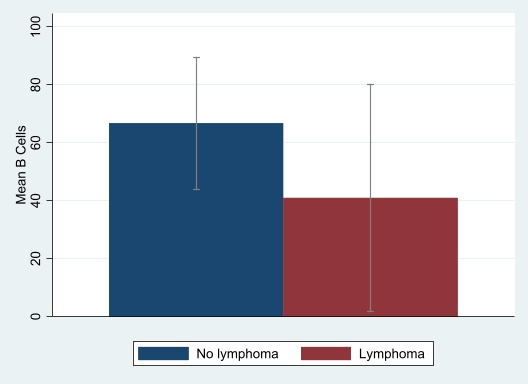

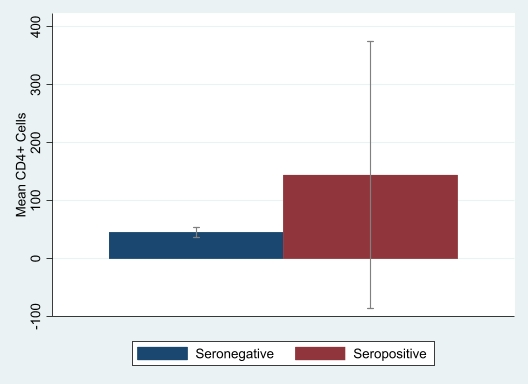

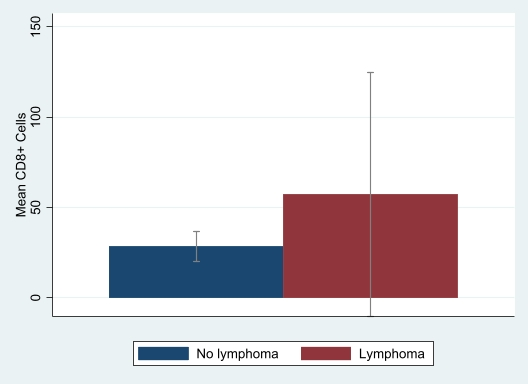

Supplement: Supplementary file 1 [file DataSheet_1.docx]
